# Reply to Spiezia et al. Comment on “Mercurio et al. Outcomes of Acute Achilles Tendon Repair with Platelet-Rich Plasma Augmentation: A Comprehensive Review of Comparative Studies. *J. Clin. Med.* 2026, *15*, 4148”

**DOI:** 10.3390/jcm15197531

**Published:** 2026-09-28

**Authors:** Michele Mercurio, Giorgia Lucia Benedetto, Elvira Immacolata Parrotta, Giovanni Cuda, Lorena Paola, Erminia Cofano, Umile Giuseppe Longo, Simone Cerciello, Giorgio Gasparini

**Affiliations:** 1 Department of Orthopaedic and Trauma Surgery, “Magna Graecia” University, “Renato Dulbecco” University Hospital, 88100 Catanzaro, Italy; michele.mercurio@unicz.it (M.M.); gasparini@unicz.it (G.G.); 2 Research Center on Musculoskeletal Health, MusculoSkeletalHealth@UMG, “Magna Graecia” University, 88100 Catanzaro, Italyerminia.cofano@studenti.unicz.it (E.C.); 3 Department of Medical and Surgical Sciences, “Magna Graecia” University, 88100 Catanzaro, Italy; 4 Department of Experimental and Clinical Medicine, “Magna Graecia” University, 88100 Catanzaro, Italy; 5 Fondazione Policlinico Universitario Campus Bio-Medico, 00128 Roma, Italy; 6 Research Unit of Orthopaedic and Trauma Surgery, Department of Medicine and Surgery, Università Campus Bio-Medico di Roma, Via Alvaro del Portillo, 21, 00128 Roma, Italy; 7 Saint Camillus International University of Health and Medical Sciences, 00131 Roma, Italy

We are grateful to the Authors for the time they dedicated to a detailed reading of our review. Their comments [1] reflect a level of scrutiny that we deeply value, and we welcome this exchange as a genuine opportunity to clarify the reasoning behind our methodological choices and to further contextualize our conclusions for the benefit of readers.

We agree that the number of comparative studies available on this topic remains limited, and that the heterogeneity across PRP preparation protocols, outcome measures, and follow-up durations is substantial, together with the limited number of comparative studies. This is the reason our manuscript was designed and labeled as a narrative review, as stated in Section 2.1, rather than as a systematic review or meta-analysis, and why no pooled statistical estimate was attempted. Our statement that PRP “may enhance early recovery” was deliberately phrased in probabilistic terms, and, as discussed in Section 4.2, we already noted that early functional improvements “were inconsistent, often derived from small sample sizes, and should be interpreted cautiously given the potential risk of type I error,” adding that such improvements “were not consistently observed across all trials and were often limited to short-term follow-ups.” We believe this language, together with the broader caveats developed throughout Section 4.3 regarding minimal clinically important differences, already reflects a cautious rather than an overstated interpretation of a genuinely heterogeneous evidence base. We nonetheless recognize that no phrasing is immune to being read more optimistically than intended, and we thank the Authors for highlighting this point.

The additional references indicated by the Authors, addressed individually below, do not alter the interpretation or conclusions of our review.

With respect to the findings of De Carli et al. [2], we note that the absence of significant between-group differences reported in that study is discussed explicitly in our manuscript in two separate Sections 3.2 and 3.3, and is further reflected in the broader synthesis of the manuscript, including the explicit statement that the study “found no differences in pain, functional scores, isokinetic performance, or imaging findings at early follow-ups.” Rather than being overlooked, this null result was presented alongside the more favorable findings of Sánchez et al. [3] and Zou et al. [4] as part of a broader narrative synthesis intended to illustrate the inconsistency that characterizes the current evidence base, consistent with the central message of our review. The Authors also highlighted additional literature that was not included in our synthesis. Regarding the PATH-2 trial reported by Keene et al. [5], we would clarify that this large and methodologically rigorous study evaluated PRP versus placebo injection within the nonoperative management of acute Achilles tendon rupture. As specified in our Eligibility Criteria (Section 2.2), our review was restricted to studies in which PRP was used as a biological adjunct to surgical repair. PATH-2 therefore addresses a related but distinct clinical question, and its exclusion reflects the defined scope of our review rather than an oversight in our search strategy; its findings may nonetheless offer useful indirect context for the nonoperative setting.

As for the meta-analyses by Boksh et al. [6] and Yang et al. [7], we are pleased that the Authors brought these valuable contributions to our attention. We would clarify that the two meta-analyses differ in their applicability: Boksh et al. pooled data from surgically treated patients, whereas the PRP-specific comparison in Yang et al. among surgically treated patients was based on only two studies and on selected outcomes, namely calf circumference ratio and ankle range of motion. Within these limits, neither analysis demonstrated a significant between-group difference in functional or biomechanical outcomes, nor did either show superiority of PRP; this absence of demonstrated superiority should not be read as proof of equivalence or as excluding any possible benefit. With this caveat, the findings are broadly consistent with the cautious position already adopted in our review, which similarly states that “current comparative evidence does not demonstrate consistent long-term clinical superiority over standard repair” and that “routine use cannot be supported based on the available data.”

We take note that the Authors agree with the clinical conclusion of our review, namely that the totality of current evidence does not support the routine clinical use of PRP as an adjunct to acute Achilles tendon repair. We remain aware, as acknowledged throughout our Discussion and Limitations sections, that the evidence base is still preliminary and heterogeneous, and that further well-designed, adequately powered trials are needed before firmer conclusions can be drawn.

We thank the Authors again for their careful engagement with our work and for the opportunity to clarify our reasoning.

We also noted that reference 1 in the Comment erroneously lists authors involved in a different, earlier study conducted by our research group [8].

In summary, we believe that the methodology we adopted is consistent and valid and that our findings are correctly reported and useful to clarify the evidence regarding PRP augmentation in the surgical repair of acute Achilles tendon rupture.

## Data Availability

No new data were created or analyzed in this study. Data sharing is not applicable to this article.
